# Theoretical Enhancement of the Goos–Hänchen Shift with a Metasurface Based on Bound States in the Continuum

**DOI:** 10.3390/mi14061109

**Published:** 2023-05-25

**Authors:** Xiaowei Jiang, Bin Fang, Chunlian Zhan

**Affiliations:** 1 College of Optical and Electronic Technology, China Jiliang University, Hangzhou 310018, China; josephjiangquzhi@126.com (X.J.); binfang@cjlu.edu.cn (B.F.); 2 College of Information Engineering, Quzhou College of Technology, Quzhou 324000, China

**Keywords:** Goos–Hänchen shift, bound states in the continuum, metasurface, reflectivity

## Abstract

The enhancement of the Goos–Hänchen (GH) shift has become a research hotspot due to its promoted application of the GH effect in various fields. However, currently, the maximum GH shift is located at the reflectance dip, making it difficult to detect GH shift signals in practical applications. This paper proposes a new metasurface to achieve reflection-type bound states in the continuum (BIC). The GH shift can be significantly enhanced by the quasi-BIC with a high quality factor. The maximum GH shift can reach more than 400 times the resonant wavelength, and the maximum GH shift is located exactly at the reflection peak with unity reflectance, which can be applied to detect the GH shift signal. Finally, the metasurface is used to detect the variation in the refractive index, and the sensitivity can reach 3.58 × 10^6^ μm/RIU (refractive index unit) according to the simulation’s calculations. The findings provide a theoretical basis to prepare a metasurface with high refractive index sensitivity, a large GH shift, and high reflection.

## 1. Introduction

The Goos–Hänchen (GH) effect was first predicted by Newton [1] and experimentally verified by the German scientists Goos and Hänchen in 1947 [2]. It occurs when a beam is totally reflected at the interface between two media. The reflected beam has a lateral shift relative to the incident beam in the incidence plane [3]. This lateral shift is known as the GH shift. The GH effect is a research hotspot due to its profound physical significance and broad application scenarios. It has been studied in various wavebands, including the visible, infrared, terahertz, and microwave wavebands [4,5,6,7]. It has also been widely used in sensors, optical switches, polarization splitting, and other fields [8,9,10].

However, without any assistance from external enhancements, the GH shift is only a few times the length of its wavelength, which is not conducive to experimental detection and practical applications. Therefore, the enhancement of the GH shift is a research hotpot at present. According to Artmann’s stationary phase method [11], the GH shift is proportional to the partial derivative of the reflection phase to the incident angle. If strong optical resonance with a high quality factor (quality factor, *Q*) could be achieved, the reflection phase would dramatically vary around the resonant angle and lead to a large GH shift. In recent years, optical resonance structures (with high *Q*) have been proposed, including photonic crystals, surface plasmon structures, metasurfaces of core–shell nanoparticles, metal gratings, and low absorption dielectric plates [12,13,14,15], which can enhance the GH shift. Although these optical resonance structures can significantly improve the GH shift, their maximum GH shift is located at the reflection dip and the reflectivity is extremely low, which makes it difficult to detect the GH shift signal.

Recently, local resonance modes in continuous bound states in the continuum (BIC) have attracted the attention of researchers. BIC is a special optical resonance state that is located in the continuous radiation state and maintains a perfect local state [16,17]. As a singularity in the continuous spectrum, an ideal BIC has infinite quality factors and the energy can be perfectly confined within the system. These characteristics greatly enhance the interaction between light and matter and are often used to reduce the laser threshold [18] and nonlinear harmonic enhancement [19]. However, due to the finite size of the structure, material absorption, and other external disturbances, the BIC tends to collapse to Fano resonance with a finite *Q* value, which is called quasi-BIC (QBIC) [20,21,22].

It has been found that reflective BIC with high reflection can be excited through asymmetric gratings, photonic crystals, metasurfaces, and other structures [23,24,25]. In addition, QBIC has an extremely high *Q* value. Thus, QBIC resonance has an ultra-narrow spectral width. This attribute tends to cause the reflection phase to change dramatically around the incidence angle, thus resulting in a large GH shift. Consequently, BIC is an effective way to enhance the GH shift. At present, many researchers such as Zhiwei Zheng [26], Feng Wu [27], and Yuhang Ruan [19] have achieved GH shift enhancement based on BIC. Zhiwei Zheng et al. utilized a silicon nanorod (suspended in air) metasurface to excite reflective BIC, and the maximum GH shift achieved was 1654 times the resonance wavelength. Moreover, the maximum value of the GH shift was located at the reflection peak with perfect reflection. Feng Wu et al. proposed the excitation of reflective BIC based on a one-dimensional dielectric asymmetric dielectric grating. The maximum GH shift was 689 times the resonance wavelength, and the wavelength of the reflectivity peak (which is close to 1) also coincided with the wavelength of the GH shift peak. Yuhang Ruan et al. proposed that by cutting a one-dimensional symmetric rectangular grating to different degrees to form an asymmetric trapezoidal grating, the switch between excitation-reflection-type BIC and QBIC could be realized. The GH shift was enhanced by 500 times the resonance wavelength, and the maximum GH shift assisted by QBIC was located at the reflectance peak with unity reflectance. However, the metasurface and grating proposed in previous works were ideal structures, which was a challenge for the existing fabrication technology. In this paper, we propose a new type of dielectric metasurface that can excite reflective resonant BIC. The BIC can be converted into QBIC with an ultra-high *Q* value through the perturbation of the metasurface structural parameters. The structure of the metasurface is simple and can be prepared with the existing fabrication technology. Our structure can produce a large phase change at the wavelength corresponding to the Fano peak, which can be used to achieve a large GH shift.

We obtain the influence of the change in metasurface structure parameters on the resonance linewidth of the reflected QBIC. Based on multiple decompositions, the internal physical mechanism of reflective QBIC resonance on the metasurface is analyzed. Finally, the GH shift of the metasurface is calculated based on the stationary phase method. The GH shift can be up to about 400 times the resonance wavelength, and the maximum GH shift is located at the reflection peak with unity reflectance. We prove that the proposed metasurface has extremely high sensitivity in refractive index detection. The sensitivity can reach 3.58 × 10^6^ μm/RIU (refractive index init), which is high enough to be applied in the fields of environmental gas and biological detection.

## 2. Device Structure and Simulation Method

**Figure 1:**
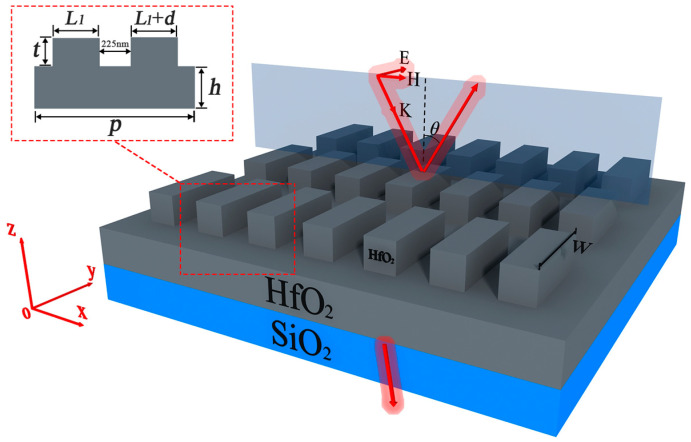
Schematic of the proposed all-dielectric metasurface.

The structure of the dielectric metasurface used to enhance the GH shift is shown in Figure 1. It can be seen from the figure that the metasurface consists of a silica (SiO_2_) substrate and hafnium dioxide (HfO_2_) rectangular nanorods, in which a layer of hafnium dioxide is embedded between the substrate and the nanorods. The designed metasurface BIC/QBIC resonance wavelength is approximately 985 nm. According to the literature [28,29], the refractive index of HfO_2_ is approximately 1.88 and the refractive index of silica is approximately 1.45. The loss in the two materials near the resonance wavelength is zero. The structural parameters of the metasurface are as follows. The thickness of the silica substrate is 10 nm, *t* = 150 nm is the thickness of the rectangular nanorod, and *h* = 50 nm is the thickness of the HfO_2_ embedded layer. *p* = 850 nm is the period of the unit cell of the metasurface, which is the same along the x-axis and y-axis, and 225 nm is the distance between the two rectangular nanorods in one unit cell along the x-axis. The two nanorods in one unit cell are arranged as follows. The lengths of the two rectangular nanorods along the y-axis are the same, which is *w* = 750 nm. The lengths of the two rectangular nanorods along the x-axis are different, at *L*_1_ and *L*_1_ + *d*, where *L*_1_ = 200 nm and *d* is the length difference.

In this research, a numerical simulation that is based on the finite-difference time-domain (FDTD) method is carried out to characterize the metasurface. The spectra as well as the electromagnetic field distributions of the metasurface presented in this paper are calculated by using the finite difference method commercial software of Lumerical FDTD solutions. The relevant incidence and boundary conditions are set as follows. The incident light is in transverse electric polarized with the electric field along the y-axis. The incident angle *θ* is defined as the angle between the incident light and the z-axis. Unless otherwise stated, note that the incident light is illuminated obliquely at an incidence angle of *θ* = 2° to the proposed structure. The Bloch boundary conditions (*θ* ≠ 0°) and periodic boundary conditions (*θ* = 0°) are added in the x-direction, periodic boundary conditions are added in the y-direction, and perfectly matched layer boundary conditions are added in the z-direction.

The metasurface shown in Figure 1 can easily be realized using current semiconductor processing technology. First, electron beam evaporation is used to deposit an HfO_2_ film on a silica substrate. Then, photolithography is performed for pattern transference. Finally, dry etching is utilized to form the patterns on the HfO_2_ layer.

## 3. Results and Discussion

### 3.1. Physical Mechanism of All-Dielectric Metasurface Excited Reflective BIC

**Figure 2:**
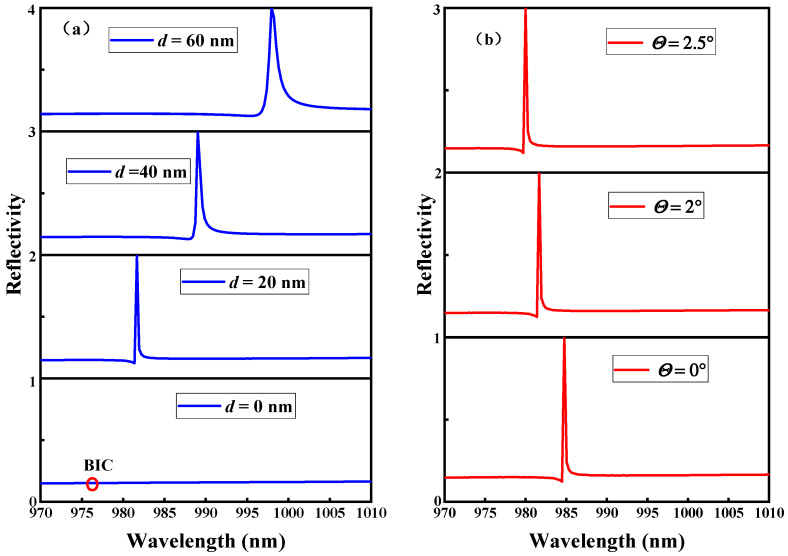
Effects of different parameters on the reflection spectrum. (**a**) Variation width *d* of the rectangular nanorods (*θ* = 2°). (**b**) Incident angle *θ* (*d* = 20 nm).

The effects of the width *d* and the incidence angle *θ* on the metasurface reflection spectrum are shown in Figure 2. As can be seen in Figure 2a, when *d* = 0 nm, because the metasurface maintains symmetry, there is no coupling channel between the metasurface and the free space. Therefore, the metasurface remains in the BIC state. Since the linewidth of the BIC is 0 and the *Q* value is infinite, the resonance cannot be seen in the reflection spectrum [20]. However, when *d* ≠ 0, i.e., when the symmetry is broken after the structural parameters of the metasurface are perturbed, the coupling channels are formed between the eigenstates of the metasurface and the free space; then, the Fano resonance with a finite *Q* value is formed in the reflection spectrum. As *d* increases, the linewidth of the Fano resonance becomes wider. As shown in Figure 2b, the linewidth of the Fano resonance does not change significantly with increasing *θ* at *d* = 20 nm.

**Figure 3:**
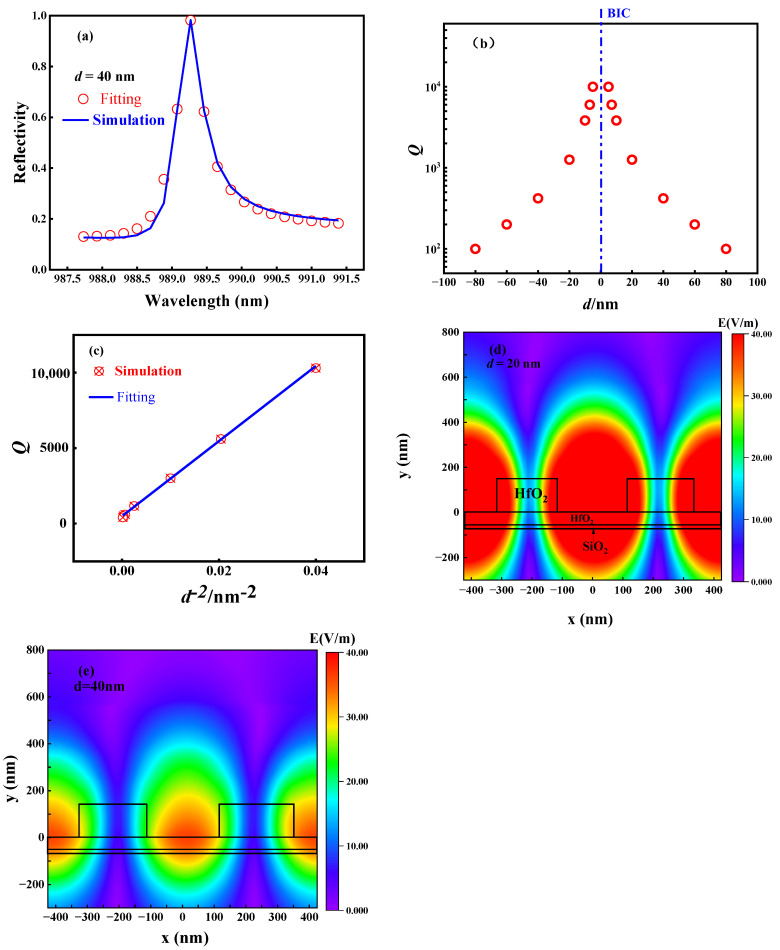
*Q* value and electric field distribution with different *d* values (*θ* = 2°). (**a**) Reflection spectrum fitting and simulation curve at *d* = 40 nm. (**b**) *Q* values with different *d* values. (**c**) Relationship between *Q* and *d*^−2^. (**d**) Distribution of the electric field on the xz plane at *d* = 20 nm. (**e**) Distribution of the electric field on the xz plane at *d* = 40 nm.

In order to understand the effect of the variation in *d* on the metasurface *Q* value, the simulated reflection spectrum needs to be fitted based on the classical Fano formula [16,30,31]. The classical Fano formula is expressed as:

(1) $$T(\omega )=T_{0}+A_{0}\frac{{\left[q+2(\omega -\omega_{0})/\Gamma \right]}^{2}}{1+{\left[2(\omega -\omega_{0})/\Gamma \right]}^{2}}$$

where *ω*_0_ is the resonant frequency, *Γ* is the resonant linewidth, *T*_0_ is the background scattering parameter, *A*_0_ is the coupling coefficient between the continuous and discrete states, and *q* is the Breit–Wigner–Fano parameter, which determines the asymmetry of the resonant spectral lines. The quality factor of the Fano resonance, *Q* = *ω*_0_/*Γ*, can be obtained from Equation (1). Figure 3a shows the fitting of the reflection spectrum of the metasurface at *d* = 40 nm using Equation (1). From Figure 3a, it can be found that the fitting curve based on Equation (1) is basically consistent with the reflection spectrum calculated with the simulation. Based on the above method, the *Q* values of the metasurface at different *d* are calculated, as shown in Figure 3b. In Figure 3b, it can be seen that when *d* is closer to 0 nm, the *Q* value is closer to infinity. When *d* = 5 nm, the *Q* value of the metasurface is approximately 1 × 10^4^. Furthermore, the metasurface *Q* value decreases with increasing *d*, which coincides with the BIC characteristics [17,23]. In Figure 3c, it can be seen that the *Q* value of the metasurface is linearly related to *d*^−2^*,* which is also consistent with the BIC-dominated asymmetric resonance theory [17,23]. Figure 3b shows that the smaller the *d* is, the larger the *Q* value is, which indicates that the smaller the energy radiated by the metasurface to the free space is, the greater the energy confined in the metasurface is, which is helpful in enhancing the interaction between the metasurface and matter when the metasurface is used as a sensor. Figure 3d,e show the electric field distribution in the xz (y = 0 nm) plane of the metasurface (*d* = 20 nm and *d* = 40 nm). By comparing Figure 3d,e, it is obvious that the energy confined in the metasurface at *d* = 40 nm is significantly less than the energy confined in the metasurface at *d* = 20 nm.

To further understand the intrinsic physical mechanism of the reflectance resonance induced by the metasurface as *d* varies, we decompose the far-field radiation of the QBIC and Fano resonance into contributions of different multipoles. The multipole moments are calculated based on the current density *j* in the metasurface cell. The specific calculation formula is shown in Equations (2)–(6) [32,33],

(2) $$P=\frac{1}{i\omega }\int jd^{3}r$$

(3) $$M=\frac{1}{2c}\int (r\times j)d^{3}r$$

(4) $$T=\frac{1}{10c}\int [(r\cdot j)r-2r^{2}j]d^{3}r$$

(5) $$Q_{}^{e}=\frac{1}{i2\omega }\int [r_{\alpha }j_{\beta }+r_{\beta }j_{\alpha }-\frac{2}{3}(r\cdot j)\delta_{\alpha ,\beta }]d^{3}r$$

(6) $$Q_{{}_{}}^{m}=\frac{1}{3c}\int [{\left(r\times j\right)}_{\alpha }r_{\beta }+{\left(r\times j\right)}_{\beta }r_{\alpha }]d^{3}r$$

(7) $$\begin{array}{l} I=\frac{2\omega^{4}}{3c^{3}}{\left|P\right|}^{2}+\frac{2\omega^{4}}{3c^{3}}{\left|M\right|}^{2}+\frac{2\omega^{6}}{3c^{5}}{\left|T\right|}^{2}+\frac{\omega^{6}}{5c^{5}}\sum_{\alpha ,\beta }{\left|Q_{}^{e}\right|}^{2}+\\ \frac{\omega^{6}}{20c^{5}}\sum_{\alpha ,\beta }{\left|Q_{}^{m}\right|}^{2}+o(\omega ) \end{array}$$

where *P*, *M*, *T*, *Q^e^*, and *Q^m^* are the moments of the electric dipole (ED), magnetic dipole (MD), toroidal dipole (TD), electric quadrupole (Qe), and magnetic quadrupole (Qm), respectively. *c* is the speed of light in vacuum, *ω* is the angular frequency, α, β = x, y, z. The scattered power of different multipoles can be obtained with Equation (7) after obtaining the multipole moments using Equations (2)–(6). In Equation (7), *I* represents the sum of the multipole scattered power, which consists of the scattered power of ED, MD, TD, Qe, Qm, and the high-order multipole (o(ω)).

**Figure 4:**
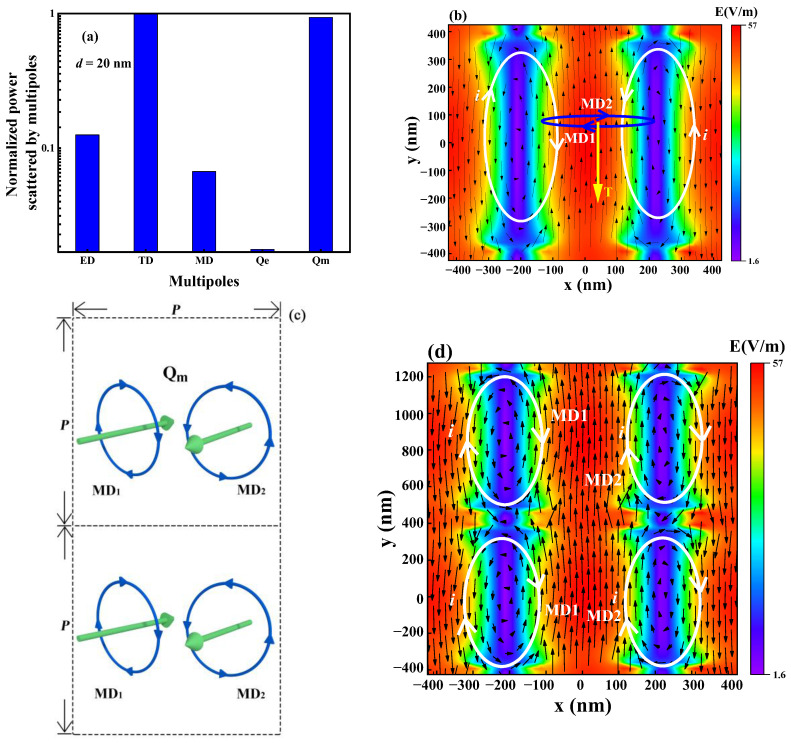
Multipole scattering power and multipole resonance formation mechanism (*d* = 20 nm, *θ* = 2°). (**a**) Scattering power of the ED, TD, MD, Qe, and Qm. (**b**) Electric field vector distribution. (**c**,**d**) Formation mechanism of magnetic quadrupole.

Figure 4a shows the multipole normalized scattering power of the metasurface at the resonance wavelength calculated with Equations (2)–(7). From the figure, it can be found that the reflection Fano resonance formed by the metasurface is the result of the joint action of ED, MD, TD, and Qm, whereas the scattering power of Qe is negligible. From the figure, it can be further deduced that the dominant resonant modes are TD and Qm, because the scattering power of TD and Qm is significantly higher than the scattering power of the other multipoles. By careful observation, it can be found that the scattering power of TD is slightly higher than the scattering power of Qm, which leads to the conclusion that the metasurface-reflected Fano resonance is the result of the coupling of the toroidal dipole and the magnetic quadrupole. Figure 4b shows the electric field distribution of the metasurface cell in the xy plane (z = 100 nm), where the black arrow indicates the electric field vector and the white circle indicates the current i (opposite to the direction of the electric field vector). From the electric field vector distribution, it can be found that there is an obvious ED [33]. It can also be seen in Figure 4b that near the two nanorods of the metasurface cell, there are two magnetic dipoles MD1 and MD2, that are reversed and connected at the beginning and end, which in turn form a toroidal dipole TD [34,35]. Since two magnetic dipoles with opposite directions are formed in each metasurface cell, the magnetic dipole distribution of the two adjacent metasurface cells is as shown in Figure 4c,d, which is a magnetic quadrupole (Qm) distribution [32]. Therefore, based on Figure 4a–c, the same conclusion can be drawn, that the reflection Fano resonance formed in the metasurface is composed of TD, Qm, ED, and MD, and TD and Qm play major roles.

### 3.2. Theoretical Calculation of GH Shift

**Figure 5:**
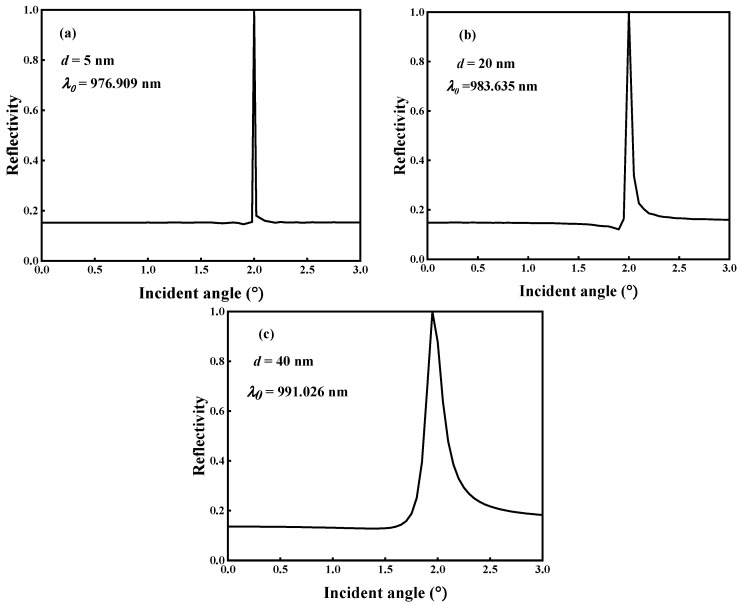
Reflection angular spectrum. (**a**) *d* = 5 nm. (**b**) *d* = 20 nm. (**c**) *d* = 40 nm.

**Figure 6:**
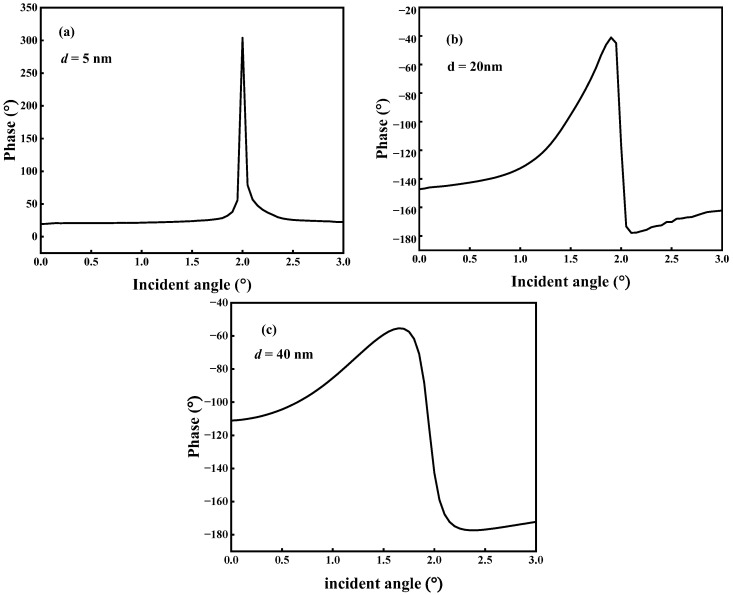
Phase angular spectrum. (**a**) *d* = 5 nm. (**b**) *d* = 20 nm. (**c**) *d* = 40 nm.

Figure 2a illustrates the QBICs with ultra-narrow resonance linewidths in the reflection spectrum that are produced by varying the metasurface geometry parameter *d*. Their corresponding reflection angular spectra (*d* = 5 nm, 20 nm, and 40 nm) are calculated by fixing the resonance wavelength *λ*_0_, as shown in Figure 5. By comparing the reflection spectra of Figure 5a–c with those of Figure 2a, it can be found that the narrower the reflection spectrum linewidth in Figure 2a is, the narrower the bandwidth of the reflection angular spectrum is, and the reflectivity also reaches 1. The reflectance phase angular spectrum was calculated for different values of *d*, as shown in Figure 6. From Figure 6, it can be seen that the linewidth of the reflectance phase angular spectrum gradually becomes wider as *d* increases. Then, based on Figure 6a–c and Artmann’s stationary phase theory [11], the GH shift of the metasurface at different *d* values is calculated. Equation (8) is the calculation formula of the GH shift when the incident beam has a sufficiently wide waist, and is expressed as:

(8) $$GH=-\frac{\lambda_{0}}{2\pi }\frac{\partial \Phi }{\partial \theta }$$

in which *Φ* is the reflection phase. From Equation (8), it can also be found that the GH shift is proportional to the partial derivative of the reflection phase to the incident angle.

**Figure 7:**
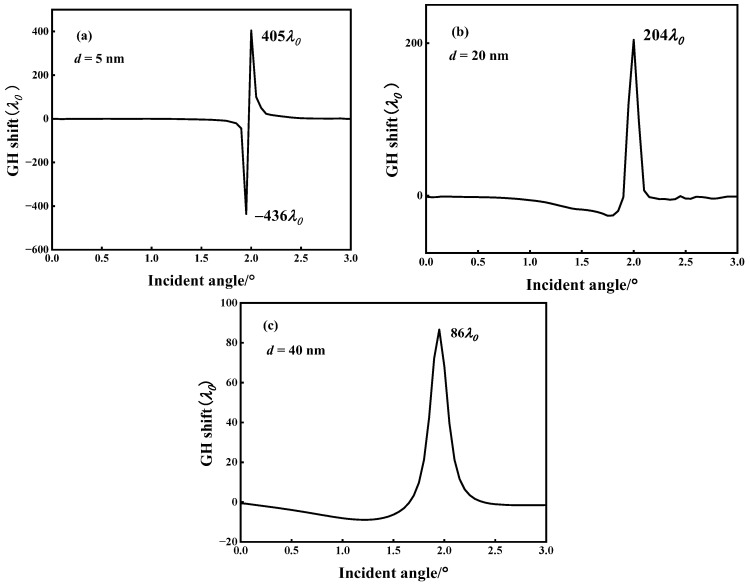
Goos–Hänchen (GH) shift angular spectrum. (**a**) *d* = 5 nm. (**b**) *d* = 20 nm. (**c**) *d* = 40 nm.

The angular spectrum of the GH shift for different *d* values is calculated based on Equation (8) and Figure 6, as shown in Figure 7. From Figure 7a–c, it can be found that the metasurface has the maximum GH shift around the incidence angle *θ* = 2°, which can be explained by Figure 2a and Figure 6; under the combined coupling of the toroidal dipole and magnetic quadrupole, the metasurface not only achieves perfect reflection resonance, but also causes the reflection phase to change the most dramatically around the incidence angle *θ* = 2°. The maximum GH shift value decreases significantly as *d* changes from 5 nm to 40 nm. When *d* = 5 nm, there are a positive maximum GH shift and a negative maximum GH shift around the incidence angle of 2°, where the positive maximum GH shift reaches 405 *λ*_0_ and the negative maximum GH shift reaches −436 *λ*_0_, as shown in Figure 7a. However, the positive maximum GH shift value around *θ* = 2° at *d* = 40 nm is only 86 *λ*_0_, as shown in Figure 7c. According to Figure 7, the GH shift can be divided into a positive GH shift and a negative GH shift. For most practical application scenarios such as sensing and wavelength division multiplexing, a positive GH shift is more desirable because the reflected light beam and the incident light beam can be effectively separated [36]. However, for some special practical application scenarios such as light storage, a negative GH shift is necessary to achieve a closed optical path [37]. In this research, we use the GH shift to detect the change in the environmental refractive index. Therefore, we focus on the analysis of the changes in the positive GH shift. Figure 5, Figure 6 and Figure 7 show that the proposed metasurface achieves significant GH shift enhancement and the maximum GH shift is located at the reflectance peak with perfect reflection, which is a significant advantage over other optical resonance microstructures that only achieve GH shift enhancement but have ultra-low reflectivity.

In general, the GH shift only occurs under the condition of total reflection because the phase of the reflected wave changes when total reflection occurs. At this time, the reflected wave travels along the direction of evanescent wave propagation for a distance and then reflects, which is the GH shift. However, the metasurface can generate and enhance GH shifts based on BIC because the metasurface can generate QBIC resonances with high *Q*. The reflection phase changes near the incident angle and can thus generate GH shifts such as total reflection conditions [38]. The high *Q* value BIC resonance of the metasurface excitation can make the reflection phase change more intensely near the incident angle compared to that of total reflection; so, it can enhance the GH shift. In Figure 7, it can be seen that the GH shift gradually decreases as *d* increases. This is because as *d* increases, the variation in the reflection phase around the incident angle slows down. As shown in Figure 6, when *d* = 5 nm, the reflection phase varies about 280° near the incident angle. However, when *d* = 40 nm, the reflected phase changes only approximately 120° near the incident angle.

**Figure 8:**
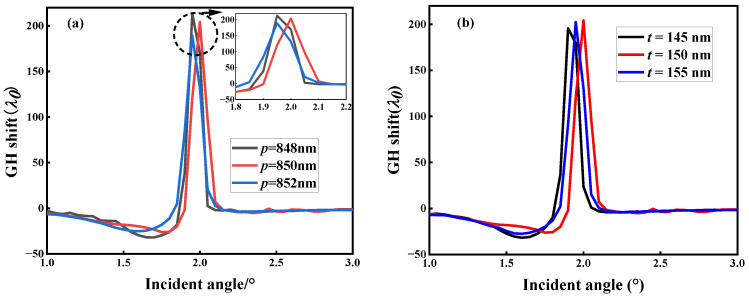
The effect of metasurface structural parameters on Goos–Hänchen (GH) shift (*d* = 20 nm). (**a**) Influence of *p* on GH shift. (**b**) Influence of *t* on GH shift.

We calculated the effects of the metasurface period *p* and rectangular nanorod thickness *t* on the GH shift, as shown in Figure 8. Figure 8a shows the effect of *p* on the GH shift. It can be seen from the figure that as *p* changes, there is no significant effect on the magnitude of the GH shift, but the angle corresponding to the maximum GH shift will shift. Figure 8b shows the effect of *t* on the GH shift. Similarly, as *t* changes, the magnitude of the GH shift remains almost unchanged, but the angle corresponding to the GH shift shows a significant shift. The changes in *p* and *t* have no significant impact on the magnitude of the GH shift, as the main determining factor affecting the *Q* value of metasurface BIC resonance is the rectangular nanorod length difference *d*. The changes in *p* and *t* do not have a significant impact on the phase of the metasurface, as they have a weaker effect on the *Q* value. Therefore, their changes have a smaller impact on the GH shift of the metasurface.

**Figure 9:**
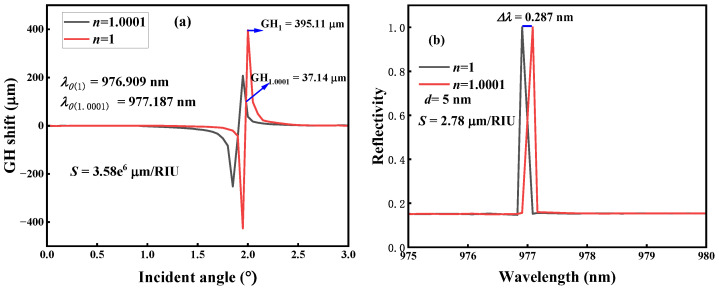
Metasurface sensor sensitivity (*d* = 5 nm). (**a**) Goos–Hänchen (GH) shift signal sensitivity. (**b**) Wavelength signal sensitivity.

From Figure 2b, it can be seen that the narrow linewidth Fano resonance formed by the metasurface has a high *Q* value when *d* ≠ 0 nm, which indicates that the metasurface has ultra-high sensitivity when used as a sensor [39]. For this reason, we apply this to refractive index (RI) sensing to detect any small changes in the environmental refractive index. RI sensors are highly required in many application scenarios such as climate monitoring and biochemical detection [40,41]. For example, carbon dioxide has an RI of about 1.0005 at a temperature of 0 °C and an atmospheric pressure of 101.325 kPa [42]. Therefore, to accurately measure the RI change in carbon dioxide, an RI sensor with a measurement accuracy of 1 × 10^−4^ is required. The metasurface proposed in this paper can meet the required measurement accuracy requirements. Figure 9 shows the changes in the GH shift and resonance wavelength (*d* = 5 nm) when the environmental RI *n* of the metasurface changes. Figure 9a shows the change in GH shift when *n* changes from 1 to 1.0001. The increase in the RI makes the maximum GH shift move toward smaller incident angles. As can be seen from the figure, when *n* = 1, the GH shift (*θ* = 2°) is 395.11 μm (the resonance wavelength *λ*_0_ (*n* = 1) = 976.909 nm). However, when *n* = 1.0001, the GH shift (*θ* = 2°) is 37.14 μm (the resonance wavelength *λ*_0_ (*n* = 1.0001) = 977.187 nm). The sensor sensitivity, which is defined as *S* = Δ*GH*/Δ*n*, is calculated to be 3.58 × 10^6^ μm/RIU, which is much higher than the sensitivity of the BIC resonance-based sensors reported in the literature [19,43,44,45,46], as summarized in Table 1. Although the metasurface proposed by Zhang et al. [24] can significantly enhance the GH shift and has an ultra-high refractive index sensitivity in the near-infrared waveband, it is suspended in air, which significantly increases the experimental preparation steps and costs; therefore, it is not included in Table 1. The metasurfaces or nanostructures listed in Table 1 are easily prepared in experiments and can excite BIC resonance. Figure 9b shows that the resonance wavelength is red-shifted when the RI *n* changes from 1 to 1.0001, and the resonance-wavelength-based sensitivity *S* = Δ*λ*_0_/Δ*n* = 2.78 μm/RIU is calculated. It can be seen from a comparison between Figure 9a,b that the signal detection using the amount of change in the GH shift as an RI sensor is nearly 1 × 10^6^ times more sensitive than that using the amount of change in the wavelength.

## 4. Conclusions

In this research, we design and simulate an excitable reflective BIC metasurface based on the finite-difference time-domain method. The metasurface can significantly improve the GH shift up to 400 *λ*_0_ due to the high *Q* value of QBIC. Compared with other GH shift enhancement approaches, the maximum GH shift of the proposed metasurface is located at the reflection peak (the reflectance is 1). The designed structure is simple and compatible with current semiconductor fabrication tecnology, which facilitates the preparation of actual devices and the detection of GH shift signals. By decomposing the far-field multipole of QBIC and Fano resonance, it is found that the ultra-narrow linewidth Fano resonance can be achieved in the metasurface by varying the *d* due to the coupling of the toroidal dipole and the magnetic quadrupole. In addition, the sensitivity of the metasurface can reach 3.58 × 10^6^ μm/RIU when the GH shift is used to feed back the change in the RI of the surrounding environment. The sensitivity is significantly improved compared to using resonance wavelength variation to reflect the RI change in the surrounding environment. This research provides a theoretical basis for the future preparation of metasurfaces with large GH shifts or a tunable GH shift with high reflectivity at the same time.

## Figures and Tables

**Table 1:** Properties of bound states in the continuum (BIC)-based metasurface sensors.

Structure	Working Waveband	Q-Factor	Refractive Index Sensitivity	Year [Reference]
1. Two silicon blocks and quartz substrate	Near-infrared waveband	6200	543 nm/RIU (simulation)	2020 [43]
2. Si-based slab, graphene layer, and quartz substrate	Near-infrared waveband	387.5	604 nm/RIU (simulation)	2021 [44]
3. Two silicon cuboids and quartz substrate	Terahertz waveband	1 × 10^5^	3 × 10^5^ μm/RIU (simulation)	2022 [45]
4. Silicon tetrameric clusters and BaF_2_ substrate	Far-infrared waveband	2000	2.75 μm/RIU (simulation)	2022 [46]
5. HfO_2_ rectangular nanorod and silicon substrate	Near-infrared waveband	1 × 10^4^	3.58 × 10^6^ μm/RIU (simulation)	This work

## Data Availability

Data are contained within the article.
